# Severe Dengue in Children: Pathogenesis, Early Recognition, and Evidence-Informed Management

**DOI:** 10.3390/tropicalmed11090252

**Published:** 2026-09-08

**Authors:** Hsien-Yi Wang, Shih-Bin Su, Chung-Yi Li, Kow-Tong Chen

**Affiliations:** 1 Department of Sport Management, College of Leisure and Recreation Management, Chia-Nan University of Pharmacy & Science, Tainan 710, Taiwan; 2 Department of Internal Medicine, Division of Nephrology, Chi-Mei Medical Center, Tainan 710, Taiwan; 3 Department of Occupational Medicine, Chi Mei Medical Center, Tainan 710, Taiwan; 4 Department of Public Health, College of Medicine, National Cheng Kung University, Tainan 701, Taiwan; 5 Department of Occupational Medicine, Tainan Municipal Hospital (Managed by Show Chwan Medical Care Corporation), Tainan 701, Taiwan

**Keywords:** dengue, children, severe dengue, pediatric dengue, early recognition, fluid management, global health

## Abstract

Severe dengue remains an important cause of pediatric hospitalization, morbidity, and mortality in tropical and subtropical regions, particularly where rapid triage and pediatric critical-care capacity are limited. This structured narrative review synthesizes the evidence on the epidemiology, pathogenesis, early recognition, diagnosis, management, and prevention of severe dengue in children. Severe disease reflects interactions among viral factors, pre-existing immunity, dysregulated host responses, and microvascular endothelial injury. Antibody-dependent enhancement, inflammatory mediators, dengue nonstructural protein 1, and endothelial glycocalyx disruption contribute to vascular hyperpermeability, plasma leakage, shock, severe bleeding, and organ impairment. Because deterioration often occurs abruptly around defervescence, serial clinical assessment, hematocrit trends, urine-output monitoring, and timely recognition of warning signs are central to risk stratification. Molecular assays and NS1 antigen testing are most useful during the early febrile phase, although diagnostic performance varies with illness timing and immune status. Carefully titrated isotonic crystalloid therapy remains the cornerstone of treatment; both delayed resuscitation and excessive fluid administration may worsen outcomes. Reducing mortality requires integrated clinical and public-health strategies combining standardized pediatric management, accessible diagnostics, effective referral systems, vaccination where appropriate, surveillance, and vector control.

## 1. Introduction

Dengue is caused by four antigenically distinct dengue virus serotypes (DENV-1 to DENV-4), which are transmitted primarily by the urban-adapted vectors *Aedes aegypti* and *Aedes albopictus* [1,2]. Its geographic range has expanded dramatically due to rapid urbanization, climate variability, vector adaptation, and increasing global population mobility [2,3,4,5,6,7]. Severe dengue associated with secondary heterotypic infection emerged as a recognized clinical phenomenon in Southeast Asia after World War II [6]. Subsequently, all four DENV serotypes spread to tropical regions of Africa and the Americas through increasing international travel and the routine movement of people, goods, and services [6,8]. Children living in hyperendemic regions are particularly vulnerable to severe plasma leakage, hemoconcentration, and dengue shock syndrome (DSS), which typically occur within a narrow and clinically unstable window during the transition from the febrile to the critical phase [9,10,11,12,13,14].

The 2009 World Health Organization (WHO) classification stratifies dengue into three categories: dengue without warning signs, dengue with warning signs, and severe dengue [1]. Severe dengue is characterized by: (1) severe plasma leakage leading to cardiovascular shock or respiratory distress; (2) severe bleeding as evaluated by the clinical team; or (3) severe organ impairment. Key warning signs—such as persistent vomiting, severe abdominal pain, mucosal bleeding, lethargy, hepatomegaly, and a rapid rise in hematocrit concurrent with a sharp decline in platelet count—serve as indispensable clinical triage tools [1]. However, these signs do not perfectly predict which pediatric patients will progress to life-threatening shock.

This review critically synthesizes the current evidence on the epidemiology, pathogenesis, early recognition, diagnosis, clinical management, and prevention of severe dengue in children. We emphasize the interface between biological mechanisms and health-system capacity, because survival depends not only on disease severity but also on rapid triage, repeated reassessment, careful fluid titration, and timely escalation of care.

## 2. Methods

We conducted a structured narrative review informed by literature searches of MEDLINE (via PubMed), Embase, Scopus, and Web of Science for English-language publications from January 2009 to June 2026. Search concepts included “severe dengue,” “pediatric” or “child,” “pathogenesis,” “endothelial dysfunction,” “warning signs,” “diagnosis,” “fluid management,” “intensive care,” and “vaccine efficacy.” We also reviewed reference lists of key articles and major guidance documents from the World Health Organization (WHO) and the US Centers for Disease Control and Prevention. We defined pediatric evidence as studies including participants younger than 18 years or reporting age-specific pediatric data. The searches were intended to identify clinically relevant and methodologically informative evidence rather than to support a formal systematic-review claim.

**Figure 1:**
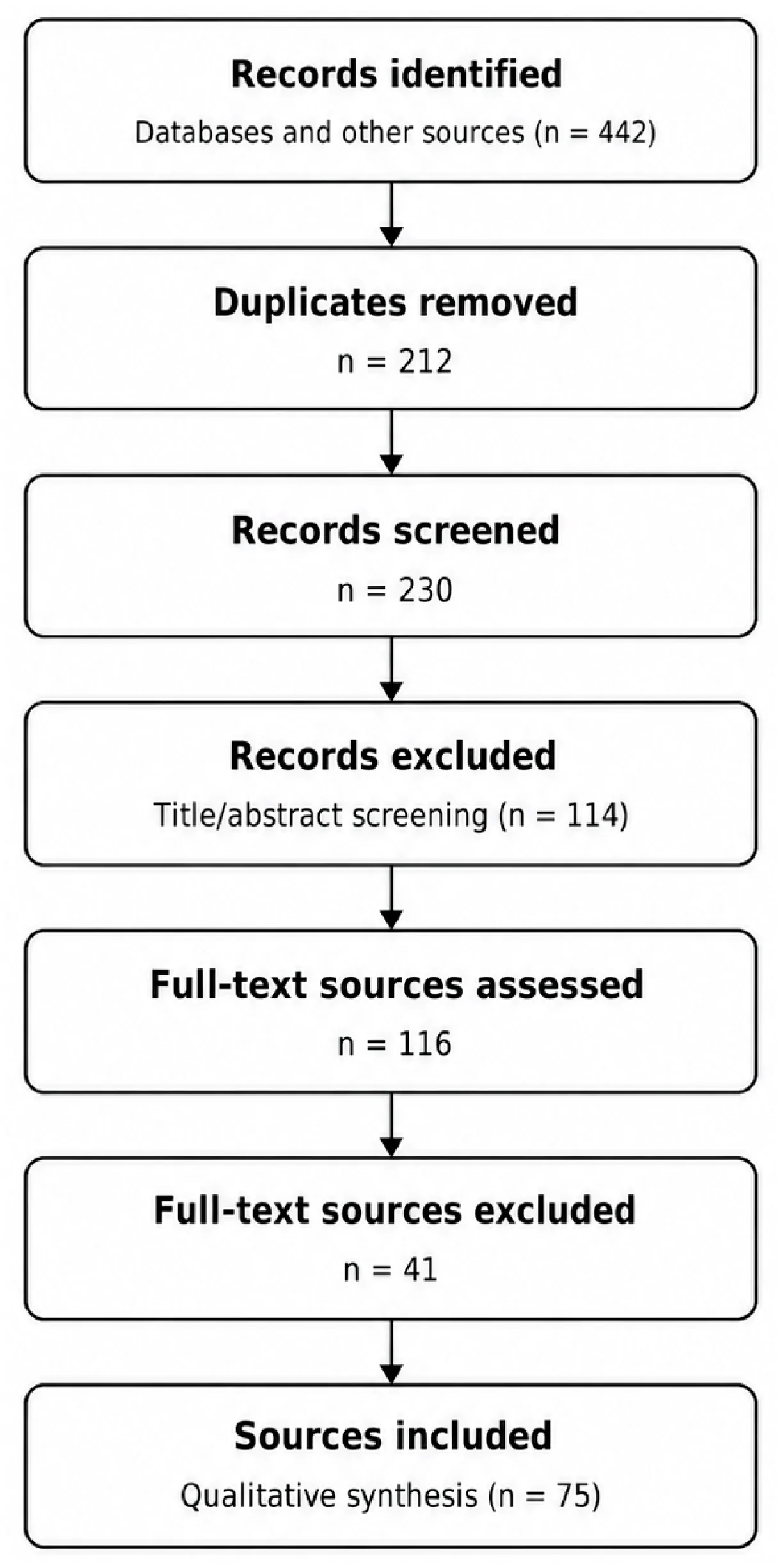
Literature identification and screening for the structured narrative review of severe pediatric dengue.

We selected evidence based on clinical relevance, methodological rigor, and applicability to pediatric dengue. We prioritized pediatric cohort studies, multicenter observational studies, randomized controlled trials, systematic reviews, and authoritative international guidelines. We defined severe pediatric dengue using the 2009 WHO criteria [1]. Given the heterogeneity in study design, setting, age definitions, and outcome measures, we synthesized findings narratively rather than pooling them quantitatively; we did not perform a formal meta-analysis or risk-of-bias assessment. We synthesized the findings thematically, emphasizing clinical translation, early recognition, management, prevention, and health-system implementation. We used PRISMA-style elements only to report literature identification and screening transparently; they should not be interpreted as a formal systematic-review methodology [15]. Figure 1 summarizes the literature-identification process. Database and other-source records identified (n = 442); duplicates removed (n = 212); records screened (n = 230); records excluded after title/abstract screening (n = 114); full-text sources assessed (n = 116); full-text sources excluded (n = 41); sources included in the qualitative synthesis (n = 75).

## 3. Epidemiology and Socioeconomic Burden

Dengue transmission has expanded far beyond historically endemic tropical regions, with rising incidences in subtropical and temperate zones [6,16,17,18]. Approximately 100 million clinically apparent infections occur annually, putting nearly half of the global population at risk [2,3]. A 10-year prospective cohort study in Vietnam, in which the median age of patients was 10 years, showed that most dengue cases were associated with secondary immune responses; all four DENV serotypes were identified, and dengue shock occurred most commonly between days 4 and 6 of illness [19]. A prospective cohort study of school-aged children in Thailand demonstrated substantial variation in the ratio of symptomatic to inapparent dengue infections (S:I ratio), both among schools within a given year and within individual schools across dengue seasons. The main determinants of the S:I ratio were dengue incidence in the current year and the preceding year [20]. In another Thai pediatric cohort, children with dengue hemorrhagic fever (DHF) more frequently had higher body temperature, nausea or vomiting, abdominal pain, rashes, diarrhea, petechiae, hepatomegaly, and lower platelet counts than those with less severe disease [21]. Recent large-scale outbreaks have severely tested the global public-health infrastructure, exposing vulnerabilities in surveillance, triage, inpatient capacity, and pediatric critical-care resources [22].

In hyperendemic regions of Southeast Asia and Latin America, co-circulation of multiple DENV serotypes drives a high pediatric disease burden, dominated by severe plasma leakage and dengue shock syndrome (DSS) [23]. Although secondary heterotypic infection is a well-documented risk factor for severe disease, severe outcomes also occur during primary pediatric infections, indicating that viral virulence, host genetics, age-related vascular physiology, and immunological history interact [12,23]. Importantly, epidemiological risk factors such as age, sex, geographic location, travel, individual history of chronic disease, vector distribution, and monitoring strategies are often derived from adult or mixed-age cohorts [24,25,26] and should not be extrapolated directly to children. In pediatric studies, younger age and obesity have been associated with severe plasma leakage or shock, although the magnitude and consistency of these associations vary across settings [6]. Regional patterns are also changing: severe pediatric disease remains prominent in Asia, whereas several Latin American settings have shifted toward older age groups as population immunity and transmission patterns evolve (Table 1) [2,3,4,5,10,11,13,27,28,29].

Severe pediatric dengue imposes substantial economic, social, and health-system burdens [30,31,32,33]. Hospitalization is a major driver of direct medical expenditure and catastrophic out-of-pocket costs in many low- and middle-income countries. Indirect costs include parental absenteeism from work and disruptions to children’s education. During seasonal epidemics, surges in pediatric admissions may overwhelm monitored beds and pediatric intensive care units, deplete blood bank reserves, and disrupt routine child health services. Although many historical estimates were based on mixed-age populations, age-stratified studies confirm that hospitalized pediatric dengue contributes substantially to household costs and dengue-related disability in endemic regions.

## 4. Pathogenesis of Severe Pediatric Dengue

The pathogenesis of severe pediatric dengue involves a multifactorial interaction among viral factors, host susceptibility, pre-existing immunity, and dysregulated inflammatory responses [34,35]. Severe disease occurs frequently, although not exclusively, during secondary infection with a heterologous DENV serotype. DHF/DSS has long been recognized in infants born to DENV-immune mothers and in individuals experiencing secondary heterotypic DENV infection [36]. During secondary heterotypic infections, sub-neutralizing or non-protective antibodies from a prior infection may facilitate viral entry into Fcγ receptor-bearing immune cells, a process termed antibody-dependent enhancement (ADE) [28,37,38,39,40]. Higher viral burden has also been associated with greater disease severity; in a pediatric cohort, each 1.0 log10 copies/mL increase in serum viral load was associated with an approximately 50% increase in the odds of severe dengue [40]. Although a transient hyperinflammatory and hypercytokinemic state may accompany severe dengue, thrombocytopenia, capillary leakage, multiorgan dysfunction, and hyperferritinemia, the extent to which cytokines directly mediate tissue injury in human dengue remains incompletely defined [41].

DENV infection begins after a bite from an infected mosquito, with initial viral replication in the skin followed by systemic dissemination through the bloodstream. Vascular leakage is a hallmark of severe dengue and plays a central role in progression to life-threatening complications, including dengue shock syndrome [42]. Circulating dengue virus nonstructural protein 1 (NS1) has been implicated in endothelial dysfunction and increased vascular permeability, although the magnitude and precise mechanisms of these effects remain under investigation [42,43]. Puerta-Guardo et al. [44] demonstrated that flavivirus NS1 can disrupt endothelial barrier integrity and promote viral dissemination across endothelial barriers in vitro and in vivo. Together, these findings support an important role for NS1-associated endothelial injury in severe dengue pathogenesis, while recognizing that endothelial dysfunction is likely multifactorial.

Endothelial glycocalyx degradation is accompanied by shedding of structural components, including syndecan-1 and hyaluronan, into the circulation [45,46,47,48]. In pediatric DSS, circulating glycocalyx and inflammatory biomarkers correlate with clinical measures of vascular leakage and hemodynamic disturbance; higher syndecan-1 levels at shock presentation have been associated with recurrent shock, although clinically actionable thresholds remain unvalidated [47]. Importantly, dengue-associated vascular permeability is usually transient and may resolve within 24–48 h as endothelial function recovers, even though the resulting intravascular volume depletion can be profound. Experimental studies indicate that NS1 can promote endothelial glycocalyx degradation through pathways involving sialidases and cathepsin L, thereby increasing vascular permeability [39,45,46].

**Figure 2:**
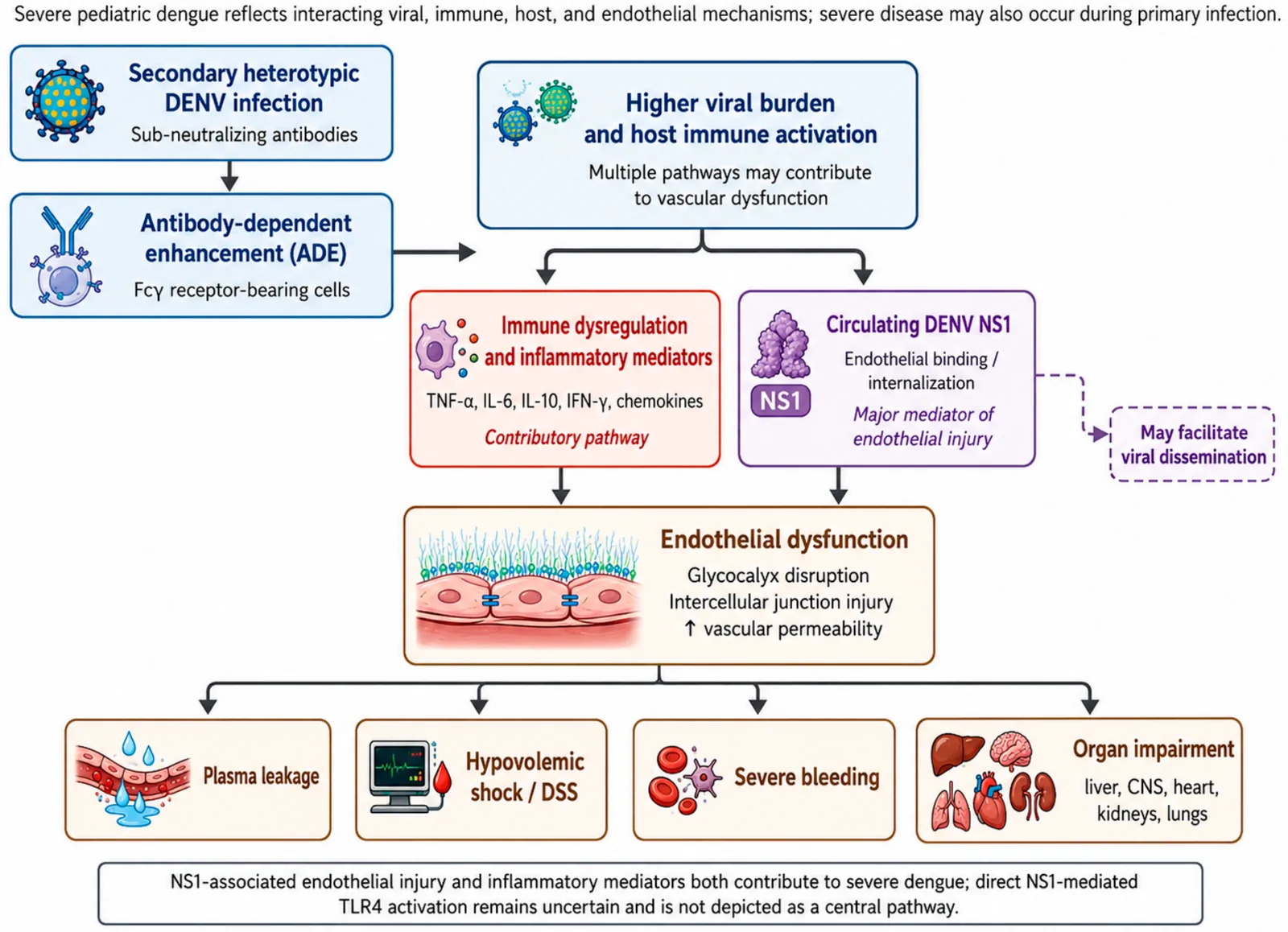
Integrative pathogenesis of severe dengue in children. Severe pediatric dengue results from interactions among viral factors, pre-existing immunity, host susceptibility, dysregulated inflammatory responses, and endothelial injury. During heterotypic secondary infection, sub-neutralizing antibodies may promote antibody-dependent enhancement and increase viral burden. Inflammatory mediators may contribute to endothelial activation, while circulating dengue virus nonstructural protein 1 (NS1) is associated with disruption of endothelial barrier integrity and the glycocalyx and may facilitate viral dissemination. These interacting mechanisms increase vascular permeability and plasma leakage, leading to hypovolemic shock, severe bleeding, and organ impairment. Severe disease may also occur during primary infection.

Figure 2 summarizes the principal pathways. Taken together, viral replication, immune activation, and endothelial injury may interact to produce severe disease. Experimental and cellular studies have demonstrated dysregulation of inflammatory mediators, including TNF-α, IFN-γ, IL-6, and IL-10 [49,50]; however, these findings should not be interpreted as establishing direct cytokine-mediated injury as the sole mechanism of severe human dengue. NS1-associated endothelial barrier dysfunction may additionally facilitate vascular leakage and viral dissemination [42,44].

## 5. Atypical and Expanded Pediatric Manifestations

Although plasma leakage and hypovolemic shock are the principal manifestations of severe pediatric dengue, clinicians should also recognize expanded dengue syndrome with severe organ involvement [51,52,53,54,55]. Neurological complications include encephalopathy, seizures, encephalitis, and, rarely, transverse myelitis; reported frequencies vary widely because they are concentrated in hospitalized and critically ill populations. Severe hepatic involvement may present with hepatomegaly, right upper quadrant pain, aminotransferase concentrations exceeding 1000 U/L, coagulopathy, or hepatic encephalopathy. Myocarditis can cause arrhythmia, ventricular dysfunction, or cardiogenic shock and may coexist with hypovolemic shock. Acute kidney injury commonly reflects prolonged hypoperfusion, although direct or immune-mediated injury may contribute. Dengue-triggered secondary hemophagocytic lymphohistiocytosis is rare but should be considered in children with persistent fever, splenomegaly, cytopenias, hypertriglyceridemia, and markedly elevated ferritin.

## 6. Early Recognition and Diagnosis

### 6.1. Clinical Recognition

Pediatric dengue typically progresses through febrile, critical, and recovery phases. Early symptoms are nonspecific and may resemble influenza, enteroviral infection, malaria, typhoid fever, rickettsial disease, or bacterial sepsis [56]. The greatest hazard occurs around defervescence, usually on illness days 3–7, when plasma leakage may begin, and clinical status can deteriorate within hours. Serial assessment is therefore more informative than a single examination. Warning signs include persistent vomiting, severe abdominal pain, mucosal bleeding, lethargy or restlessness, hepatomegaly (>2 cm), oliguria, cold or clammy extremities, delayed capillary refill, narrowing pulse pressure, and a rising hematocrit accompanied by a rapidly falling platelet count. Severe bleeding is less common than plasma leakage in children but may be occult; it should be suspected when hemodynamic instability persists despite apparently adequate fluid replacement [57].

### 6.2. Laboratory Confirmation and Diagnostic Gaps

Early laboratory confirmation supports triage and outbreak control, but each diagnostic method has limitations. Reverse-transcription polymerase chain reaction (PCR) is highly specific and sensitive during the early febrile phase, generally on illness days 1–5, but its availability is limited in many settings, and sensitivity declines as viremia falls [58,59]. NS1 antigen assays provide a rapid and relatively inexpensive alternative; however, sensitivity is lower in secondary infection, partly because pre-existing antibodies form immune complexes that reduce detectable circulating antigen [60]. IgM and IgG assays are more useful later in illness but may cross-react with other flaviviruses and with vaccine-induced antibodies [61]. Plaque-reduction neutralization testing can support serotype-specific assessment and serosurveillance but is technically demanding, expensive, and too slow for routine acute management [62].

The practical diagnostic objective is not merely to confirm dengue, but to identify children likely to deteriorate and to exclude conditions requiring different treatment. Test results should therefore be interpreted in the context of illness duration, prior infection, vaccination history, local epidemiology, and serial clinical findings.

## 7. Clinical Management

Supportive care and carefully titrated fluid therapy are the foundations of management. Children with shock, respiratory distress caused by plasma leakage, severe bleeding, or organ impairment require immediate hospital care and close monitoring, ideally where pediatric critical-care support is available [55,63]. Isotonic crystalloids, such as 0.9% saline or Ringer’s lactate, are first-line fluids for shock. The bolus volume and infusion rate should be individualized based on perfusion, pulse pressure, mental status, urine output, serial hematocrit, and response to initial treatment. Fluid requirements should be reassessed frequently and reduced promptly when perfusion improves or the recovery phase begins.

A multicenter retrospective analysis of 691 children with dengue shock syndrome in Southeast Asia found that mixed crystalloid-colloid strategies were associated with greater respiratory compromise, more ventilatory support, and longer hospitalization than crystalloid-only resuscitation after adjustment for baseline severity [64]. Although residual confounding cannot be excluded, these findings support reserving synthetic colloids for carefully selected patients with refractory shock despite appropriately titrated crystalloid therapy.

### Managing the Fluid Paradox

The central therapeutic challenge is the “fluid paradox”: inadequate resuscitation prolongs shock, whereas excessive fluid during the leakage phase can cause pulmonary edema, respiratory failure, and tense ascites, particularly when extravasated fluid is reabsorbed during recovery [55,56,63]. Intravenous fluids should therefore be adjusted dynamically using repeated assessment of perfusion, pulse pressure, urine output, respiratory status, and hematocrit trends. Where available, point-of-care ultrasound may help identify pleural effusion, ascites, or evolving volume overload, and lactate could support assessment of tissue hypoperfusion. In resource-limited settings, consistent use of simplified pediatric fluid algorithms and basic bedside monitoring is likely to provide the greatest scalable benefit. No antiviral or immunomodulatory treatment has yet shown definitive efficacy in large clinical trials; meticulous supportive care and avoidance of iatrogenic fluid overload remain the priorities.

Clinically significant bleeding or persistent hemodynamic instability despite adequate volume replacement should prompt evaluation for occult hemorrhage and consideration of red-cell transfusion. Prophylactic platelet transfusion is discouraged because platelet count alone does not reliably predict bleeding and transfusion has not demonstrated clinical benefit [63].

## 8. Prevention and Control

### 8.1. Pediatric Vaccination

Achieving balanced protection against all four DENV serotypes without increasing the risk of severe disease in immunologically naive recipients is the central challenge in dengue-vaccine development. CYD-TDV (Dengvaxia) is recommended only for individuals with evidence of previous DENV infection, because vaccination of seronegative recipients can increase the subsequent risk of hospitalization and severe dengue [65].

TAK-003 (Qdenga) is a live-attenuated tetravalent vaccine. Long-term phase III data show sustained protection against virologically confirmed and hospitalized dengue, but efficacy varies by serotype and baseline serostatus, with the strongest evidence for DENV-1 and DENV-2 and uncertainty regarding DENV-3 and DENV-4 in seronegative recipients [66,67,68].

WHO recommends programmatic use of TAK-003 in children aged 6–16 years in settings with high dengue transmission intensity and substantial disease burden. Countries are advised to use local age-specific seroprevalence and dengue-hospitalization data to guide introduction, with targeted subnational implementation where transmission is heterogeneous. WHO does not currently recommend programmatic use in low-to-moderate transmission settings because the efficacy-risk profile for DENV-3 and DENV-4 in baseline-seronegative recipients remains insufficiently defined. Vaccine introduction should be accompanied by risk communication, safety surveillance, and long-term effectiveness monitoring [69].

### 8.2. Integrated Prevention Framework

Vaccination should complement, not replace, established public-health measures [70,71,72]. An integrated prevention framework combines vector surveillance and control, environmental management, community participation, timely diagnostics, outbreak preparedness, and, where appropriate, vaccination. Wolbachia-infected mosquito deployment has reduced dengue incidence in a cluster-randomized trial and represents a promising component of integrated vector management [72].

## 9. Health-System and Global Health Implications

Severe pediatric dengue tests health-system preparedness. Delayed referral, inconsistent triage, limited laboratory access, shortages of monitored beds, and unequal access to pediatric intensive care widen preventable mortality gaps [63,70]. During outbreaks, tertiary centers may become overwhelmed precisely when frequent reassessment and careful fluid adjustment matter most.

The most scalable priorities are standardized pediatric triage and fluid protocols, repeated frontline training, dependable referral pathways, equipment-light monitoring, and integrated clinical-surveillance data. Research should validate prognostic tools that add value beyond routine measures, define pediatric thresholds, and evaluate implementation across different resource settings [62,71].

## 10. Discussion

This review highlights three clinically important messages. First, severe pediatric dengue is primarily a syndrome of transient, immune-mediated endothelial dysfunction and glycocalyx injury rather than direct viral cytopathology alone. Second, the central clinical task is to recognize the transition to plasma leakage and provide sufficient, carefully measured resuscitation while avoiding fluid overload. Third, the effectiveness of clinical guidance depends on health-system capacity, including trained frontline staff, rapid laboratory support, clear referral criteria, and resilient pediatric critical-care services during epidemic surges.

Several areas of clinical uncertainty remain. Although secondary infection is a major risk factor, it does not fully predict clinical outcome. Host genetics, viral genotype, immune imprinting, and prior exposure to other flaviviruses may modify pediatric susceptibility to severe plasma leakage. Biomarkers such as syndecan-1, hyaluronan, and IL-10 provide mechanistic insights, but their incremental prognostic value and clinically actionable thresholds remain undefined for routine pediatric care [6,47,48].

Although WHO warning signs remain indispensable for triage, their imperfect specificity may contribute to over-triage and strain referral centers during large outbreaks [1]. Future research should validate pragmatic pediatric prognostic models that combine serial clinical observations with widely available measurements, such as hematocrit, platelet count, and lactate, rather than relying on a single novel biomarker. The clinical value of point-of-care ultrasound also requires evaluation through standardized training, quality assurance, and outcome-based implementation studies.

Finally, health systems must adapt to the challenges of climate change, rapid urbanization, and population mobility, which are expanding dengue transmission into new regions [73]. Hospital and pediatric ICU preparedness cannot be substituted by vector-control or vaccination programs alone. Conversely, clinical excellence cannot offset unmitigated viral transmission. A coordinated approach linking pediatric vaccination, active vector management, real-time epidemiological surveillance, and strengthened pediatric referral networks represents the most sustainable path to reducing the global burden of severe pediatric dengue [71]. Table 2 summarizes the integrated framework of the mechanisms and clinical and global health implications of severe pediatric dengue [2,3,4,5,7,46,61,63,71,74,75].

## 11. Conclusions

Severe dengue in children results from interactions among viral factors, pre-existing immunity, dysregulated inflammation, and microvascular endothelial injury, and deterioration may abrupt occur around defervescence. Repeated clinical assessment, early recognition of plasma leakage, carefully titrated isotonic crystalloid therapy, prompt identification of bleeding or organ dysfunction, and timely escalation of care can minimize mortality. Future priorities include validating pragmatic pediatric prognostic tools, evaluating point-of-care monitoring strategies, refining pediatric fluid thresholds, and conducting implementation research across resource-diverse settings. At the population level, accessible diagnostics, continuous frontline training, equitable referral pathways, vaccination in appropriate high-transmission settings, real-time surveillance, and integrated vector control should be advanced together rather than as isolated interventions.

## Figures and Tables

**Table 1:** Geographic Heterogeneity in Pediatric Dengue Epidemiology and Clinical Burden.

Region	Predominant Age Group	Transmission Pattern	Key Clinical/Epidemiological Features	References
Southeast Asia (e.g., Vietnam, Thailand, Philippines)	Children (<15 years)	Hyperendemic; perennial with seasonal peaks.	High prevalence of secondary heterotypic infections; severe plasma leakage (DSS) is a primary hallmark in pediatric cohorts.	[2,3,4,5,11,12,19,20]
Latin America (e.g., Brazil, Colombia, Mexico)	Shifting toward young adults.	Cyclical epidemics; increasing endemicity.	Significant upward shift in median age over the last decade; children still face the highest mortality risk during outbreaks.	[2,3,14,27,28]
South Asia (e.g., India, Sri Lanka)	Mixed (all age groups).	Expanding urban/peri-urban transmission.	Co-circulation of DENV-1–4; rapid surge in pediatric hospitalizations within expanding urban centers.	[2,3,29]
Sub-Saharan Africa	Incompletely defined.	Sporadic outbreaks; high undiagnosed burden.	Frequent misdiagnosis as malaria or sepsis; true disease burden is likely underestimated due to surveillance gaps.	[2,3,5]

**Table 2:** Integrated Framework of Severe Pediatric Dengue: Mechanisms, Clinical Impact, and Global Health Imperatives.

Principal Domain	Key Mechanisms & Findings	Clinical Consequences	Global Health Implications
Pathogenesis	Antibody-Dependent Enhancement (ADE) and Immune Dysregulation: Sub-neutralizing antibodies may enhance viral entry and increase viral burden; inflammatory responses may further contribute to endothelial dysfunction.	Systemic Plasma Leakage: Rapid progression to hypovolemic shock and multiorgan dysfunction.	Biomarker Access: Urgent need for affordable, point-of-care prognostic markers in resource-limited settings.
Endothelial Integrity	Endothelial Barrier and Glycocalyx Injury: DENV NS1-associated mechanisms and host inflammatory responses may disrupt endothelial barrier integrity and the glycocalyx.	Fluid Management Paradigm: Narrow therapeutic window; high risk of iatrogenic fluid overload during recovery.	Protocol Standardization: Necessity of universal, pediatric-specific fluid algorithms across all healthcare tiers.
Clinical Phenotype	Age-dependent Variation: Children primarily manifest plasma leakage, whereas adults more frequently exhibit hemorrhage.	Abrupt Deterioration: Clinical course transitions rapidly, typically during the critical phase (defervescence).	Triage Resilience: Strengthening primary care screening to prevent the collapse of tertiary systems during outbreaks.
Prevention & Diagnostics	Immune Imprinting: Vaccine efficacy (e.g., CYD-TDV vs. TAK-003) is modulated by prior infection history.	Diagnostic Gaps: Reduced NS1 sensitivity in secondary infections and flaviviral serological cross-reactivity.	Equitable Access: Ensuring fair vaccine distribution and robust post-licensure safety monitoring in low- and middle-income countries (LMICs).

## Data Availability

This study did not create or analyze new data. Data sharing is not applicable to this article.
